# Social Coping by Masking? Parental Support and Peer Victimization as Mediators of the Relationship Between Depressive Symptoms and Expressive Suppression in Adolescents

**DOI:** 10.1007/s10964-012-9782-7

**Published:** 2012-06-28

**Authors:** Junilla K. Larsen, Ad A. Vermulst, Rob Eisinga, Tammy English, James J. Gross, Elin Hofman, Ron H. J. Scholte, Rutger C. M. E. Engels

**Affiliations:** 1 Behavioural Science Institute, Radboud University Nijmegen, P.O. BOX 9140, 6500 HE Nijmegen, The Netherlands; 2 Department of Psychology, Stanford University, Stanford, CA USA

**Keywords:** Depression, Emotion regulation, Social support, Peers, Parents

## Abstract

Expressive suppression is regarded as a generally ineffective emotion regulation strategy and appears to be associated with the development of depressive symptoms among adolescents. However, the mechanisms linking suppression to depressive symptoms are not well understood. The main aim of this study was to examine two potential mediators of the prospective relationship from depressive symptoms to expressive suppression among adolescents: parental support and peer victimization. Structural equation modelling was used to construct a three-wave cross-lagged model (*n* = 2,051 adolescents, 48.5 % female, at baseline; 1,465 with data at all three time points) with all possible longitudinal linkages. Depressive symptoms preceded decreases in perceived parental support 1 year later. Decreases in parental support mediated the relationship between depressive symptoms and increases in expressive suppression over a 2-year period. Multi-group analyses show that the mediation model tested was significant for girls, but not for boys. No evidence for other mediating models was found. Although initial suppression preceded increases in depressive symptoms 1 year later, we did not find any evidence for the reversed link from suppression to depressive symptoms. Clear evidence for a reciprocal relationship between depressive symptoms and parental support was found. However, only limited and inconsistent support was found for a reciprocal relationship between depressive symptoms and peer victimization. Finally, although some evidence for a unidirectional relationship from parental support to increases in suppression was found, no significant prospective relationship was found between peer victimization and suppression. The implications of our clear results for parental support, and mostly lacking results for peer victimization, are discussed.

## Introduction

People sometimes regulate their emotions *after* an emotional response has been activated, by inhibiting the behavioural display of emotion (Gross 1998). This emotion regulation strategy has been termed *expressive suppression*. The habitual use of expressive suppression is regarded as a generally ineffective strategy, because it does not reduce the experience of negative emotion and has physiological (e.g., increased cardiovascular activation), social (e.g., lower social support, less closeness to others), and cognitive (e.g., impaired memory functioning) costs (Butler et al. 2003; Gross 1998; Richards and Gross 2000; Srivastava et al. 2009). Yet, despite the importance of expressive suppression, there has been surprisingly little focus on the development of this emotion regulation strategy.

Cross-sectional studies showing a positive association between expressive suppression and depressive symptoms in adults (Gross and John 2003; John and Gross 2004) and adolescents (Betts et al. 2009; Hsieh and Stright in press) have often been interpreted as reflecting the impact of expressive suppression on depressive symptoms. However, in a recent two-wave longitudinal study among adolescents, we found that depressive symptoms preceded increased use of suppression, while suppression did not precede future depressive symptoms (Larsen et al. in press). This suggests that a unidirectional relationship exists between expressive suppression and depressive symptoms and sheds light on a framework for understanding the development of suppression during the adolescent years. Identifying mechanisms linking depressive symptoms to suppression among adolescents is an essential next step in developing theory-based interventions targeting processes that can explain why depressive symptoms may lead to this generally ineffective emotion regulation strategy.

The present three-wave longitudinal study is a follow-up of our previous two-wave study (Larsen et al. in press) and aimed to extend our initial work suggestive of a unidirectional relationship from depressive symptoms to expressive suppression. The mechanisms underlying this association are not well understood. The main purpose of the current investigation was to address this gap in the literature by examining two potential mediators of the prospective relationship from depressive symptoms to expressive suppression among adolescents: parental support and peer victimization. We considered a conceptually based model with all possible longitudinal linkages. As such, our study adds to the few previous studies testing bidirectional associations between depressive symptoms and relationship variables, and is the first to examine bidirectional associations between relationship variables (i.e., parental support and peer victimization) and expressive suppression. All possible intervening models following from the longitudinal linkages found in this study were tested.

## Theoretical Background on Mediating Models

Theoretically, emotion regulation may fulfil different functions, including supporting specific goal pursuits and satisfying hedonic needs (Koole 2009). Thus, although expressive suppression is generally ineffective at regulating the experience of emotion, it may serve other purposes. Expressive suppression can be regarded as a goal-oriented strategy, which is driven by people’s beliefs and potentially influenced by abstract theories that people have about emotion regulation (Koole 2009). There is some evidence among adults suggesting that depressed people judge their negative emotions as less socially acceptable than do non-depressed people, and that appraising one’s emotions as unacceptable mediates the relationship between negative emotion intensity and use of suppression (Campbell-Sills et al. 2006). Although evidence among adolescents is lacking, it is possible that adolescents with depressive symptoms also appraise their emotions as unacceptable.

Increased use of suppression may be a goal-oriented response to problems within close relationships (such as parent–child) and abstract theories that adolescents have about the unacceptability of expressing negative emotions. Inherently transactional interpersonal theories of depression (e.g., Coyne 1976; Coyne et al. 1991) postulate that individuals’ behaviors related to displaying negative affect (e.g., irritability, excessive reassurance seeking, corumination) elicit rejection and stress in their close relationships, which may further exaggerate depressive symptoms. In line with these theories, recent work among adolescents provides evidence for the idea that co-rumination (the excessive discussion of problems with close others) is one transactional process that connects internalizing problems and interpersonal stressors over time (Hankin et al. 2010). Adolescents are increasingly metacognitive and aware of what others are thinking of them. If adolescents are aware of the interpersonal problems following from their excessive discussion of problems, they may attempt to inhibit their display of negative emotions, and might thus shift from openness to masking their expression of emotions. Moreover, some research has shown that victimized youth develop increased maladaptive avoidant coping strategies, and that they may do so in an attempt to prevent additional victimization (Hampel et al. 2009; Puhl and Joerg 2012). Thus, trying not to show emotions may also be a goal-oriented attempt to prevent additional victimization among adolescents who experience depressive symptoms. In sum, there is reason to expect that adolescents with depressive symptoms who experience lacking parental support or peer victimization might increase their goal-directed use of expressive suppression.

In addition to this goal-oriented function, suppression also might be regarded as a need-oriented emotion regulation strategy, regulating the overt display of emotion to promote satisfaction of the need to minimize negative emotion. This is in line with Campbell-Sills and Barlow’s (2007) emotion dysregulation theory, which states that individuals with depressive symptoms tend to avoid their emotions, with this avoidance limiting emotional self-disclosures. Previous research among adults suggests that expressive suppression may be used in attempts to alter or avoid undesirable thoughts and feelings (Kashdan et al. 2006). It is unknown whether the same may apply to adolescents. However, it is possible that adolescents with depressive symptoms may use suppression directly to manage their depressive symptoms. They might be more likely to do so when they experience lacking parental support or peer victimization. Adolescents with depressive symptoms who experience lacking parental support or peer victimization have to deal with additional undesirable thoughts and feelings because of the interpersonal stressors they experience. Moreover, the fever model of normal emotion regulation suggests that self-disclosure is a curative factor in recovering from distress (Stiles 1987), and adolescents are less likely to disclose to parents with whom relationships are not satisfying (Finkenauer et al. 2004; Hare et al. 2011). Thus, adolescents with depressive symptoms who experience lacking parental support might be more likely to seek non-sharing ways (e.g., suppression) to regulate their negative feelings. In sum, adolescents with depressive symptoms who experience lacking parental support or peer victimization also might increase their use of suppression because of the need-oriented function of this strategy to minimise negative cognitions and feelings.

Based on general transactional models of development (e.g., Sameroff & MacKenzie, 2003) and transactional interpersonal theories of depression (e.g., Coyne, 1976; Coyne et al., 1991), we considered a conceptually based model with all possible longitudinal linkages. As such, all possible intervening models were tested. That is, we tested not only models built on our initial work suggestive of a unidirectional relationship from depressive symptoms to expressive suppression (Larsen et al. in press) but also models considering the reversed relationship from suppression to depressive symptoms. Suppression may also influence interpersonal stressors and longer-term depressive symptoms. Increased use of expressive suppression may lead to an inability to communicate emotional experiences to important others, resulting in a lack of social support for managing future depressive symptoms (Keenan et al. 2009). Although suppression did not precede depressive symptoms in our two-wave study (Larsen et al. in press), it is possible that suppression is linked with longer-term depressive symptoms through lacking parental support.

## Empirical Evidence for Longitudinal Linkages

### Parental Support

Few previous studies have examined the potential reciprocal relationship between depressive symptoms and parental support, and those that do exist provide mixed findings. Young et al. (2005) did not find evidence for any pathway; that is, parental support did not precede changes in depressive symptoms, and depressive symptoms did not precede changes in parental support. A few studies reported significant effects of parental support on later depressive symptoms, but no effects of depressive symptoms on parental support over time (Sheeber et al. 1997; Stice et al. 2004), while other studies provided evidence for bidirectional pathways (Branje et al. 2010; Needham 2008; Slavin and Rainer 1990). Notably, two of the more recent studies supporting bidirectional pathways were large population-based studies (Branje et al. 2010; Needham 2008), which bolsters the confidence that can placed in these findings. We thus expected to find reciprocal relationships between depressive symptoms and parental support.

The potential bidirectional relationship between parental support and expressive suppression has not been examined. Research supports the idea that lower (parental) support is associated with suppressing emotions (Graham et al. 2008; Srivastava et al. 2009). Moreover, one longitudinal study showed that the use of expressive suppression preceded less parental support over time among college students (Srivastava et al. 2009). However, this study did not test for bidirectional associations. Poorer parent–child relationships have been shown to precede higher levels of secrecy from parents in adolescence (Keijsers et al. 2010), and a strong link between the verbal and behavioral suppression of emotion has been established among adults (Kahn et al. 2012). Although adolescent studies on the link between the verbal and behavioural suppression of emotion are lacking, there is thus reason to expect that poorer parent–child relationships and low perceived parental support also may precede an increased use of expressive suppression among adolescents.

### Peer Victimization

Peer victimization is strongly associated with depressive symptoms (Hawker and Boulton 2000), and prospective research suggests that the relationships between peer victimization and depressive symptoms are likely reciprocal in nature (Hodges and Perry 1999; McLaughlin et al. 2009; Vernberg 1990). We thus expected to find reciprocal relationships between peer victimization and depressive symptoms. To date, the associations between peer victimization and expressive suppression have yet to be examined. Prior adult research suggests that suppression is not related to evaluative impressions (Gross and John 2003). For instance, among college students, suppression was not related to likability over time (Srivastava et al. 2009). Considering the negative relationship between likability and peer victimization among adolescents (de Bruyn and Cillessen 2010), we expected that suppression would *not* lead to victimization over time. However, considering that victimized youth with depressive symptoms may use suppression as a tool to avoid negative emotions or to prevent additional victimization, we expected victimization to precede increased use of suppression.

## Moderating Role of Gender

Girls exhibit a greater relational orientation and value interpersonal connectedness more than boys (Cross and Madson 1997; Rose and Rudolph 2006). Girls’ relational orientation may increase their vulnerability to lacking (parental) support. As such, they might be more likely to develop depressive symptoms in response to lacking parental support and might also make greater efforts than boys to avoid lacking parental support by means of suppression. Our study is the first to examine the prospective relationship from parental support to suppression. Previous studies have shown mixed findings with respect to the gender-specific link from parental support to depressive symptoms. Some studies found that this link was stronger for girls (Leadbeater et al. 1999; Slavin and Rainer 1990), while others did not (Meadows et al. 2006; Needham 2008). No evidence was found for any gender differences in the relationship from peer victimization to depressive symptoms among adolescents (Bakker et al. 2010; Hodges and Perry 1999; McLaughlin et al. 2009). This may be explained by the fact that peer victimization may correspond to perceptions about victimization by peers in general, with whom interpersonal connectedness may or may not play a role.

## The Current Study

The main aim of the present study was to test whether parental support and peer victimization act as mediators in the relationship between depressive symptoms and subsequent use of expressive suppression. Drawing on theory and past research showing that depressive symptoms precede lower parental support (Branje et al. 2010; Needham 2008) and more peer victimization (Hodges and Perry 1999; Vernberg 1990), as well as increased use of expressive suppression (Larsen et al. in press), we hypothesized that these relationship variables would mediate the link between depressive symptoms and prospective elevations in expressive suppression. The mediating effect for parental support was expected to be stronger for girls, as girls may make greater efforts than boys to avoid lacking parental support by means of suppression, given their greater relational orientation (Cross and Madson 1997; Rose and Rudolph 2006). Based on general transactional models of development (e.g., Sameroff & MacKenzie, 2003) and transactional interpersonal theories of depression (e.g., Coyne, 1976; Coyne et al., 1991), we considered a conceptually based model with all possible longitudinal linkages. Possible moderating effects of gender were also examined. Based on theory and (initial) past research, we expected the following longitudinal linkages. First, a unidirectional relationship was expected from depressive symptoms to suppression. Second, reciprocal relationships were expected between depressive symptoms and parental support (with the link from support to depressive symptoms being stronger for girls than for boys), and between parental support and expressive suppression. Finally, although we also expected reciprocal relationships between depressive symptoms and peer victimization, a unidirectional relationship was expected from victimization to suppression. All possible intervening models following from the longitudinal linkages found in the current study were tested. Overall, the current study applies a powerful test of mediation using a longitudinal design with three separate assessments, controlling for pre-existing and concurrent associations (Masten et al. 2005).

## Method

### Participants and Procedure

Data were collected in three waves with one-year intervals. Participants were recruited from 7 randomly selected secondary schools in suburban (*n* = 3) and urban (*n* = 4) areas from three regions in the Netherlands. Data collection took place at schools. A total of 90 classes (on average 13 per school) participated, with an average size of approximately 25 students per class. Participants were informed that participation was voluntary and confidential. Parents were informed about the study through the mail, and were asked to respond via telephone or email if they did not want their child to participate in the study. Of the 2,216 students targeted, 92.6 % (*n* = 2,051) initially participated: 85.5 % of these participants (*n* = 1,753) also completed surveys at time 2, and 76.7 % (*n* = 1,574) completed surveys at time 3. In total, 71.4 % (*n* = 1,465) of the adolescents completed surveys at all three time points. All participants attended regular secondary education and were in either first or second grade (equivalent to Grades 7 and 8 in the United States) at baseline (mean baseline age = 13.8, SD = 0.7). Boys (*n* = 741) and girls (*n* = 724) were approximately equally represented. At the beginning of the study, 18.6 % attended low secondary education, 19.7 % intermediate secondary education, 25.0 % intermediate to high secondary education, and 35.4 % attended the highest level of secondary education possible in the Netherlands. Most of the participants were born in the Netherlands (95.9 %), had at least one parent who was born in the Netherlands (94.1 %), and were living with both parents in intact, non-divorced families (89.2 %). From the ethnic minorities (10 %), 2.2 % were Turkish, 0.8 % Moroccan, 0.7 % were Surinam, Antillean or Aruban, and 6.3 % had a different ethnic background.

A logistic regression analysis comparing adolescents that participated three times and those that dropped out at one or two time points showed that attrition was predicted significantly by age (OR = .80, *p* < .05, 95 % CI = .65–.98), ethnicity (OR = 1.58, *p* < .01, 95 % CI = 1.19–2.11), family structure (OR = 1.46, *p* < .01, 95 % CI = 1.10–1.93), education level (OR = 0.91, *p* < .01, 95 % CI = 0.85–0.97), and depressive symptoms (OR = 1.03, *p* < .001, 95 % CI = 1.01–1.04). Females, younger adolescents, those of Dutch origin, those living with two biological parents, those with higher education, and those with lower levels of depressive symptoms were overrepresented in the longitudinal sample; no differences were found between drop-outs and completers for expressive suppression. However, the Cox and Snell indicator of total explained variance was .04, suggesting that the predictor variables explained limited variance in attrition.

### Measures

#### Depressive Symptoms

The Dutch version of the Center for Epidemiological Studies Depression (CES-D) inventory was used to measure depressive symptoms. The CES-D (Radloff 1977) is a 20-item self-report scale originally developed to measure depressive symptoms in the general population. Participants used a four-point response format to indicate how frequently in the past week each depressive symptom had occurred. The CES-D has shown good internal consistency and test–retest reliability among (Dutch) adolescent populations (e.g., Cuijpers et al. 2008; Roberts et al. 1990). Cronbach’s *α* in the current study was .88 at time 1, .87 at time 2, and .88 at time 3.

#### Expressive Suppression

Expressive suppression was assessed with a four-item scale of the Emotion Regulation Questionnaire, ERQ (Gross and John 2003). The ERQ expressive suppression scale has shown good reliability, consistent evidence of unifactorial structure, and convergent and discriminant validity in both younger and older adults. An example of an ERQ suppression item is: “I control my emotions by not expressing them.” In accordance with prior research on adolescents, we used a 5-point rating scale instead of the 7-point rating scale used for adults (Gullone et al. 2010). Adolescents completed the questionnaire in the presence of a researcher (or graduate student) and could ask questions about any unclear items. Cronbach’s Cronbach’s *α* was .69 at time 1, .76 at time 2, and .75 at time 3.

#### Parental Support

Parental support was measured with a brief 12-item version of the Relational Support Inventory (RSI; Scholte et al. 2001) for support perceived from fathers and mothers combined. The items tapped several aspects of emotional and instrumental support. Example items include: ‘My parents let me know that they love me’ and ‘My parents support me’. Answers were rated on a 6-point-scale (ranging from 1 = never to 6 = always). Higher scores indicated higher levels of support. Cronbach’s *α* was .84 at time 1, .83 at time 2, and .85 at time 3.

#### Peer Victimization

Peer victimization was assessed with a question from the Dutch version of the Olweus Bully/Victim Questionnaire (Olweus 1989; Solberg and Olweus 2003). This is a well-documented and validated questionnaire. In the questionnaire, we first provided a clear definition of what is meant by victimization. We defined victimization as follows: “We can say a student is being victim of bullying when another student or a group of peers says malicious or hurtful things to him. The same is true when a student is being hit, kicked, threatened, or is being excluded from the group. These things can be classified as bullying when they happen frequently or regularly, and when it’s difficult for the student being bullied to defend him or herself. It is NOT bullying when two or more students who are equally strong tease each other or fight with each other”. Being bullied was assessed with the question, “How often did other children bully you in this school year?”. Adolescents could answer with the following options: “I am not bullied,” “one or two times,” “I am regularly bullied,” “about once a week,” or “several times a week.” Options for answering the item were slightly modified from the original questionnaire: the original category “2 or 3 times a month” was changed to “I am regularly bullied.” Following the convention recommended by Solberg and Olweus (2003), this one item question was dichotomized. Students who reported not being bullied or only one or two times were classified as “not victimized” (coded as 0), whereas those who reported being regularly bullied or more often were classified as “victimized” (coded as 1). Measuring victimization with this one (dichotomized) item has been done in many previous (Dutch) studies on bullying (e.g., Branson and Cornell 2009; Fekkes et al. 2006; Giletta et al. 2010) and is considered a valid way of dividing adolescents into victims and nonvictims (Solberg and Olweus 2003).

### Statistical Analyses

#### Descriptive Analyses

Analyses were performed with PASW statistics 18. Changes over time and sex differences for the model variables depressive symptoms, expressive suppression and parental support were examined by repeated measures analyses of variance with sex as between-subject factor. For the binary variable victimization we applied repeated measures logistic regression within the Generalized Estimating Equations (GEE) module. We assumed the binomial distribution as underlying probability distribution (suited for binary variables) and used the binary logistic link function to obtain a linear relationship with the predictors time and sex.

#### The Cross-Lagged Models

**Fig. 1:**
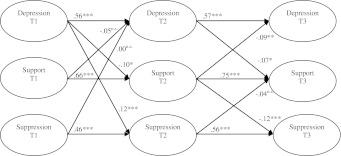
Cross-lagged model with parental support. χ^2^ (634) = 1623.87, *p* = .000, CFI = .969 and RMSEA = .028

To examine associations of depressive symptoms, parental support and suppression over time we constructed a three-wave cross-lagged model, see Fig. 1 (model 1). The three variables were included as latent variables. The control variables gender, age, ethnicity and educational level were linked to all the nine variables of the model. The structural equation model was estimated with Mplus 5.0 (Muthén and Muthén 1998–2007).

There were a large number of items for depressive symptoms (20) and parental support (12), so there would be a large number of parameters to be estimated in the structural equation model, decreasing the power of the parameter estimates of the model. For this reason, the items of these two latent variables were replaced by parcels. A parcel is (in our case the mean of) a subset of items. Depressive symptoms were measured by 4 parcels, each parcel consisted of 5 items. Parental support was also represented by 4 parcels, each parcel representing 3 items. To allocate items to parcels we used the “item-to construct balance” method as described in Little et al. (2002). The standardized single factor solution of depressive symptoms (and of parental support) at T1 was used and the item with the highest factor loading was allocated to the first parcel, the item with the second highest factor loading to parcel 2, the item with the third highest loading to parcel 3 and the item with the fourth highest loading to parcel 4. The next four item were allocated to the parcels in a reversed order (the item with the fifth highest loading to parcel 4, with the sixth highest loading to parcel 3, with the seventh highest loading to parcel 2 and with the eighth highest loading to parcel 1. This procedure is repeated for the remaining items. In this way, each parcel reflect the factor structure of the latent variable in a more or less equivalent way (see also Huver et al. 2007; Van den Eijnden et al. 2010). Identical parcels were formed at T2 and T3. The original four items of the latent variable suppression were used as indicators. In the cross-lagged model error terms of identical indicators over time were correlated (cf. Finkel 1995).

Prior to the analyses of the cross-lagged model we tested the measurement part of the first model using Confirmatory Factor Analysis. A factor model with nine latent variables and four indicators for each latent variable was tested. The factor model showed a very good fit with χ^2^ (516) = 1,141.62, *p* = .000, CFI = .980 and RMSEA = .024. Factor loadings varied from .54 to .87 indicating that the indicators reflect the 9 latent variables very well. This means that the measurement part of the structural equation model in Fig. 1 adequately fits the data. The three-wave cross lagged model of Fig. 1 is also used to examine the role of victimization. The latent variable support is replaced by the manifest variable victimization (model 2).

#### Model Fit and Missing Values

Model fit is reported by χ^2^ (*df*), *p* value and two fit measures: (a) the Root Mean Square Error of Approximation (RMSEA) (Byrne 1998) and (b) the Comparative Fit Index (CFI) of Bentler (1990). A good fit is indicated by values below .05 for the RMSEA and above .95 for the CFI. To account for missing values (14.5 % missing respondents at T2 and 23.3 % at T3), we used the Full Information Maximum Likelihood (FIML)-estimator (which uses all available information in the data) to test model 1 with support as latent variable. For model 2 with victimization as manifest two-categorical variable (victimized vs. not victimized) the Weighted Least Square parameter estimator with standard errors and Mean- and Variance-adjusted Chi-square test statistic (WLSMV)-estimator was used. This estimator is developed for ordered categorical variables. The problem of missing values for this estimator is solved by using all available pairwise information in the data.

#### Testing Cross-Paths, Mediation and Moderation

A reciprocal effect means that the cross paths between a pair of two latent variables over time (e.g., depression and support from T1 to T2, see Fig. 1) are not significantly different. Differences between cross-paths in the first cross-lagged model were tested using the Chi-square difference test. The Chi-square of the final cross-lagged model was compared with the same model including the two cross paths constrained to be equal. A significant Chi-square difference is an indication that the two cross paths have different regression weights. Almost the same procedure applies to Model 2. Because this model has a manifest binary indicator for victimization, the WLSMV-estimator was used. For this estimator, Mplus has a built-in DIFFTEST to test differences.

Mediating effects were tested as indirect effects. An indirect effect is the product of the incoming and outgoing path of the mediator. The standard error of this indirect effect is estimated in Mplus according to the delta method as described in Mackinnon et al. (2002). The moderating effect of gender in Model 1 was tested by multiple group analysis. In Mplus the factor loadings and intercepts of identical latent variables for boys and girls are constrained to be equal. The Chi-square of this baseline model was compared with the Chi-square of the same model but now included with constrained paths across gender. The latter means that each of the stability paths (the horizontal paths in Fig. 1) and each of all possible cross paths were constrained to be equal across gender. A significant difference between the baseline Chi-square and the constrained Chi-square is an indication that one or more paths have different regression weights. If this is the case, post hoc testing with Bonferroni correction were used to detect which of the paths are significant. Bonferroni correction means that the usual critical *p* value of .05 will be divided by 18 (i.e., the total number of stability paths and cross paths). This critical value (.003) will be used for post hoc testing. The moderating effect of gender in Model 2 is tested in the same manner but now using the DIFFTEST of Mplus.

## Results

### Descriptive Analyses

Table 1 shows the means and standard deviations for depressive symptoms, expressive suppression, and parental support at the three time points for boys and girls separately. Girls reported higher overall levels of depressive symptoms than boys (F = 138.52, *p* < .001), and boys reported higher levels of expressive suppression than girls (F = 39.08, *p* < .001). There were no differences between boys and girls on parental support (F = 0.99, *p* = 32). Boys showed a significant higher level of victimization (.05) than girls (.03) with Wald χ^2^ (1) = 10.57, *p* = .001 (see Table 1). Almost 16 % of the adolescents reported at least some depressive symptoms (using CES-D ≥16) and nearly 6 % of these reported moderate to severe depressive symptoms (CES-D ≥24) at T1. These percentages remained about equal over time (i.e., across T1, T2 and T3) and, there was not a significant time change for depressive symptoms across the three waves, *F*(2, 1,433) = 1.79, *p* > .10. A significant time effect was found for parental support, *F*(2, 1,308) = 22.69, *p* < .001, implying that adolescents experienced decreased parental support over time. Victimization also showed a significant decrease over time from .06 (T1) to .04 (T2) and .02 (T3) with Wald χ^2^ (1) = 29.21, *p* = .000. Finally, a significant time effect was found for expressive suppression, *F*(2, 1,432) = 3.28, *p* < .05, such that suppression decreased across over time. However, there was a marginally significant time × sex interaction (*p* = .08), suggesting that the decrease in suppression mainly applied to boys.

**Table 1:** Means and SD for depressive symptoms, expressive suppression, parental support, and victimization at baseline (time 1), 1 year later (time 2), and 2 years later (time 3) for boys and girls

	Boys		Girls	
	M	SD	M	SD
Depressive symptoms				
Time 1	7.03	5.77	10.27	8.37
Time 2	6.64	5.69	10.29	8.13
Time 3	6.34	5.94	10.25	8.24
Expressive suppression				
Time 1	10.10	3.18	9.12	3.16
Time 2	10.04	3.26	9.11	3.39
Time 3	9.69	3.15	9.10	3.31
Parental support				
Time 1	4.98	0.59	5.01	0.63
Time 2	4.89	0.61	4.94	0.69
Time 3	4.89	0.60	4.90	0.67
Victimization				
Time 1	.07	.26	.05	.21
Time 2	.06	.23	.02	.16
Time 3	.03	.18	.02	.13

* *p* < .001

Table 2 shows the correlations between the main model variables. Depressive symptoms were associated with more expressive suppression and less parental support at all time points for both boys and girls. Moreover, parental support was negatively associated with expressive suppression on all time points. This correlation between support and suppression was stronger for girls than for boys at T2 (Fisher test: Z = 3.20, *p* < .01) and T3 (Fisher test: Z = 2.94, *p* < .01).

**Table 2:** Correlations between all main variables for boys and girls separately

		1	2	3	4	5	6	7	8	9	10	11	12
1	Depressive symptoms T1	–	**.55**	**.47**	**.27**	**.28**	**.20**	**−.39**	**−.37**	**−.33**	**.22**	.14	**.21**
2	Depressive symptoms T2	**.47**	–	**.57**	**.21**	**.36**	**.23**	**−.30**	**−.42**	**−.41**	**.18**	**.16**	.08
3	Depressive symptoms T3	**.36**	**.51**	–	**.22**	**.25**	**.35**	**−.30**	**−.34**	**−.39**	**.18**	.10	**.25**
4	Expressive suppression T1	**.24**	**.13**	**.08**	–	**.46**	**.36**	**−.22**	**−.19**	**−.22**	**.22**	.09	.07
5	Expressive suppression T2	**.24**	**.26**	**.16**	**.36**	–	**.51**	**−.23**	**−.31**	**−.30**	.11	**.21**	.02
6	Expressive suppression T3	**.19**	**.19**	**.21**	**.33**	**.47**	–	**−.14**	**−.30**	**−.33**	**.26**	.07	.08
7	Parental support T1	**−.38**	**−.20**	**−.14**	**−.16**	**−.13**	**−.13**	–	**.62**	**.60**	**−**.04	**−**.12	**−**.11
8	Parental support T2	**−.26**	**−.30**	**−.20**	**−.12**	**−.16**	**−.15**	**.54**	–	**.73**	**−**.12	**−**.16	**−**.08
9	Parental support T3	**−.27**	**−.28**	**−.30**	**−.16**	**−.15**	**−.21**	**.54**	**.61**	–	−**.19**	**−**.19	.00
10	Victimization T1	**.36**	**.16**	**.16**	**.08**	.02	**−**.00	**−.13**	.06	**−**.04	–	**.66**	**.31**
11	Victimization T2	**.24**	**.22**	**.19**	**−**.04	.10	**−**.02	.04	.08	.03	**.61**	–	**.42**
12	Victimization T3	**.22**	**.26**	**.31**	.08	.01	.12	**−**.01	.01	**−**.11	**.72**	**.80**	–

Above the diagonal for girls; below the diagonal for boys. Correlations between variables 1–9 are Pearson correlations and between variables 10–13 tetrachoric correlations. Correlations of variables 1–9 with variables 10–12 are biserial correlations. Correlations in bold are significant with at least *p* < .05

Gender, age, ethnicity and educational level were moderately correlated with some variables of the cross-lagged model and were therefore used as covariates. Gender was positively related to depressive symptoms T1 (β = .23, *p* < .001), T2 (β = .15, *p* < .001) and T3 (β = .10, *p* < .001) and with suppression T1 (β = −.19, *p* < .001) and T2 (β = −.11, *p* < .001). Age was negatively associated with parental support T1 (β = −.09, *p* < .001). Ethnicity was positively related to depressive symptoms T1 (β = .09, *p* < .001) and suppression T1 (β = .10, *p* < .001). Educational level was negatively related to depressive symptoms T1 (β = −.09, *p* < .001). Girls showed more depressive symptoms than boys (T1, T2 and T3) and experienced less suppression (T1, T2). Older adolescents experienced less parental support (T1). Ethnic minority groups showed more depression and suppression at T1. Higher levels of educational level showed lower levels of depressive symptoms (T1).

### The Cross-Lagged Models

The results of the cross-lagged Model 1 (model with parental support) are presented in Fig. 1. All possible paths between the latent variables from T1 to T2 and from T2 to T3 were tested in one analysis. Non-significant paths are not shown in the model except if one of two cross paths were significant. The fit of the model was good with CFI >.95 and RMSEA <.05. The link from suppression T1 (T2) to depressive symptoms T2 (T3) was not significant. A significant cross-path was found from depressive symptoms T1 to suppression T2 but not from T2 to T3 (nor from T1 to T3). When both paths from T1 to T2 were constrained to be equal there was a significant decrease of model fit: χ^2^ (1) = 10.71, *p* = .001. This result indicates that from T1 to T2 the link from depressive symptoms to suppression was dominant.

Significant consistent cross-paths were found from depressive symptoms T1 (T2) to parental support T2 (T3): more depressive symptoms at T1 (T2) was associated with less parental support at T2 (T3). The link from parental support to depressive symptoms was significant from T2 to T3 (more parental support T2 was associated with less depressive symptoms T3), but not from T1 to T2. Equating the cross-path from parental support T2 to depressive symptoms T3 to be equal to the cross-path from depressive symptoms T2 to parental support T3 gave a non significant increase in Chi-square χ^2^ (1) = 0.68, *p* = .410. Similarly, the difference in Chi-square between the unconstrained and the constrained model was not significant from T1 to T2: χ^2^ (1) = 2.74, *p* = .098. These results indicate similar bidirectional associations between parental support and depressive symptoms (none of the paths appeared to be dominant).

A significant cross-path was also found from parental support T2 to suppression T3 (less parental support at T2 was associated with more expressive suppression at T3). This relationship was not found from T1 to T2. Moreover, none of the links from suppression to support were significant. Equating the cross-path from parental support T2 to suppression T3 to be equal to the cross-path from suppression T2 to parental support T3 showed a significant decrease of model fit: χ^2^ (1) = 7.46, *p* = .006. This result indicates that the relationship from parental support to suppression was dominant between T2 and T3.

Cross-lagged Model 2 (with parental support of Model 1 replaced by peer victimization) showed a good model fit with χ^2^ (345) = 1014.77, CFI = .955 and RMSEA = .031. Only one significant cross-path was found: victimization at T2 was related to more depressive symptoms at T3, β = .17, *p* < .001. Equating the cross-path from depressive symptoms T2 to victimization T3 to be equal to the cross-path from victimization T2 to depressive symptoms T3 (β = .13, *p* > .10) gave a non significant increase in Chi-square: χ^2^ (1) = 2.96, *p* = .086, indicating a bidirectional (not dominant) relationship between victimization and depressive symptoms from T2 to T3.

### Testing Mediation

In Model 1 the link from depressive symptoms T1 to parental support T2 and from parental support T2 to suppression T3 were both significant. Testing mediation showed that the indirect link from depressive symptoms T1 via parental support T2 to suppression T3 was significant with z = 2.44, *p* = .015. Because the links from depressive symptoms T1 to victimization T2 and from victimization T2 to suppression T3 in Model 2 were not significant, testing mediation is superfluous. Also, no possible other mediating models could be tested.

### Moderating Effects of Gender

Multiple group analysis was used to test moderating effects of gender. In Model 1 no overall significant difference between boys and girls was found with χ^2^ (18) = 15.99, *p* = .593. However, the indirect path from depressive symptoms T1 via parental support T2 to suppression T3 was significant for girls (z = 2.30, *p* = .021) but not for boys (z = .64, *p* = .523). In Model 2 we found an overall significant difference between boys and girls with χ^2^ (18) = 33.13, *p* = .016. However, post hoc testing with Bonferroni corrected alpha showed that no paths were significantly different between boys and girls.

## Discussion

Recently, there is increasing attention for expressive suppression in the developmental literature (Betts et al. 2009; Chambers et al. 2009; Gullone et al. 2010; Hsieh and Stright in press). However, there has been surprisingly little focus on the development of this emotion regulation strategy. The present three-wave longitudinal study is a follow-up of our previous two-wave study (Larsen et al. in press) and aimed to extend our initial work suggestive of a unidirectional relationship from depressive symptoms to expressive suppression. The mechanisms underlying this association are not well understood. The main purpose of the current investigation was to address this gap in the literature by examining two potential mediators of the prospective relationship from depressive symptoms to expressive suppression among adolescents: parental support and peer victimization. We considered a conceptually based model with all possible longitudinal linkages. As such, our study adds to the few previous studies testing bidirectional associations between depressive symptoms and relationship variables (e.g., Branje et al. 2010; McLaughlin et al. 2009), and is the first to examine bidirectional associations between relationship variables (i.e., parental support and peer victimization) and expressive suppression. Overall, this large study of adolescents extends the literature on emotion regulation and psychological adjustment by providing insight into the unfolding of depressive symptoms, relationship variables (i.e., parental support and peer victimization), and expressive suppression over time. We used a longitudinal design with three separate assessments, which allowed us to control for pre-existing and ongoing concurrent associations and test models of bidirectional influences from one domain of adaptation to another (Masten et al. 2005).

The results can be summarized as follows. First, the present study further supports our initial work (Larsen et al. in press) suggestive of a unidirectional relationship from depressive symptoms to increased use of expressive suppression. We did not find any evidence for the reversed relationship from suppression to depressive symptoms. Second, our study provides generally consistent evidence supporting reciprocal negative associations between depressive symptoms and parental support, while less consistent support was found for a bidirectional association between depressive symptoms and peer victimization. Third, our study is the first to provide longitudinal evidence documenting the prospective relation between parental support, but not peer victimization, and subsequent use of expressive suppression. Related to the most central question of this investigation, as hypothesized, decreased parental support emerged as an intervening variable in the relationship from depressive symptoms to increased use of expressive suppression, but this mediation effect only applied to girls. In contrast to our expectations, there was no evidence for a similar mediating role of peer victimization, or for other possible intervening models. The effect sizes of the relationships found in the current study were small, but consistent with previous literature. Overall, our findings provide novel evidence consistent with the idea that parental support, but not peer victimization, is a mechanism explaining why girls who experience depressive symptoms report increased use of expressive suppression over time.

### Mediating Model

Our mediation findings suggest that depressive symptoms in girls increased the risk of expressive suppression use over 2 years through the mechanism of decreased parental support, rather than that it effected expressive suppression per se. A zero-order direct effect is not a prerequisite for mediation (Zhao et al. 2010). It might be that competitive underlying mechanisms operate simultaneously, inducing non-significant direct effects. For instance, youths with continuing depressive symptoms use mental health care services at a higher rate (Schraedley et al. 1999), and at mental health care services youth probably express their depressive problems. Simultaneously, they may suppress their verbal and behavioural display of emotion specifically in response to decreased parental support. So there may be contextual effects such that depressive symptoms may lead adolescents to suppress more around people they do not feel supported by, but there is not a consistent overall effect on the direct habitual use of suppression over 2 years.

No evidence was found for a mediating role of peer victimization in the depression-suppression relation. Not only did depressive symptoms not significantly precede later peer victimization, peer victimization also showed no significant associations with expressive suppression. Although it is possible that relationships with peers really do not explain the depression-suppression relation, we suggest it is more likely that this null mediation finding is due to the specific measure of peer relationships that we used: if we measured peer support (instead of victimization) we expect that we would have found mediation by peers as well. Peer victimization corresponded to perceptions about victimization by peers in general (who may or may not be friends or important people in the lives of victimized adolescents). Thus, close interpersonal mechanisms may be more important in explaining why girls with depressive symptoms increase their use of expressive suppression. Future research should test both peer and parental support as mediators.

### Moderating Effects of Gender

We expected that parental support would play a stronger mediating role in the link from depressive symptoms to suppression for girls than for boys. However, we found that parental support *only* mediated the effect on suppression for girls. In contrast to our hypothesis, the negative prospective relationship from parental support to subsequent use of expressive suppression did not differ for boys versus girls, nor did any of the other relationships. It should be noted that the cross-sectional association between parental support and expressive suppression was stronger for girls than for boys at two time points. Thus, significant prospective moderation by gender may have been found if the constructs were lagged at a shorter term within a 1 year time frame. Nevertheless, it might seem counterintuitive that our intervening model only applied to girls, while gender did not moderate any of the established longitudinal associations. This may be explained as follows. Girls exhibit a greater relational orientation (Cross and Madson 1997; Rose and Rudolph 2006). Thus, girls with depressive symptoms, compared to boys with depressive symptoms, may be more focused on their underlying co-ruminating behaviours preceding reduced support (Hankin et al. 2010), and might respond by suppressing their display of emotion. This reasoning might support a goal-oriented function of suppression. However, it is also possible that girls with depressive symptoms who experience decreases in support use suppression as a need-oriented strategy to manage depressive symptoms. Girls self-disclose more than boys (Papini et al. 1990; Rose and Rudolph 2006) and might thus have a greater need to find “replacement” of self-disclosure as a curative strategy in managing depressive symptoms (Stiles 1987) after experiencing problems with sharing feelings (e.g., lacking parental support). Future research may provide more insight into these possible dynamic processes proposed if multi-informant methods and approaches, such as observations and a dynamic systems approach, are employed (Granic and Hollenstein 2003).

### Parental Support

Our study adds to the few previous studies testing reciprocal longitudinal models that can provide insight into the direction of effects between parental support and adolescent depressive symptoms. As hypothesized, we found evidence for reciprocal associations between depressive symptoms and parental support. These findings are in line with previous incidental results testing bidirectional relationships between parent–child supportive relationships and depressive symptoms (Branje et al. 2010; Needham 2008) and are consistent with research highlighting the bidirectional nature of associations among other parenting factors and adolescent depressive symptoms (Hamza and Willoughby 2011). Overall, this supports transactional models of reciprocal parent–child relationships.

The most consistent evidence was found for the path from depressive symptoms to parental support. There are various mechanisms that may explain this relationship from depressive symptoms to parental support. Following interpersonal theories of depression (e.g., Coyne 1976; Coyne et al. 1991), individuals who experience depressive symptoms often persistently seek reassurance since they discount the positive feedback that they obtain from close others, such as parents. Close others subsequently begin to feel frustrated since they are unable to minimize the insecurities of their child with depressive symptoms (Evraire and Dozois 2011). Thus, parents may see themselves as lacking power and respond by disengagement or behaving negatively toward their adolescent children (Bugental et al. 1999; Shields and Beaver 2011). In addition, through excessive reassurance seeking, the distress and desperation of a person with depressive symptoms also may be transmitted from the child to the parent (Joiner and Katz 1999), with parental depressive symptoms known to impact on parenting quality and parental support provided (Lovejoy et al. 2000). Finally, it may be that the perception of adolescents who experience depressive symptoms are biased, reflecting the tendency to increasingly interpret their environment in a negative way (Beck 2005), such that they think their parents support them less but parents actually do not provide less support. Future research should examine the mechanisms underlying the finding that depressive symptoms were linked consistently with deterioration in perceived parental support.

Our study was the first to examine the bidirectional relationship between parental support and expressive suppression. Although we expected bidirectional associations, we only found some evidence that parental support preceded the development of increased expressive suppression. No evidence was found for the reversed path, that is, from expressive suppression to parental support. In contrast, Srivastava et al. (2009) found that stable expressive suppression preceded lower future social support from parents among college students; however, this study did not test for bidirectional associations so it’s possible that parent support also would have also preceded future use of suppression in this college sample. It might be that the negative effects of the habitual use of suppression on social support mainly become apparent during later adolescence or early adulthood. Future research should test this by tracking both suppression and indicators of social functioning across many points in time across adolescence and young adulthood.

### Peer Victimization

We expected a reciprocal relationship between peer victimization and depressive symptoms during adolescence. We found that more peer victimization significantly preceded the development of depressive symptoms at one time point, and that the reversed, non-significant, relationship from depressive symptoms to peer victimization did not differ from the established significant pathway. This provides some limited support for a reciprocal relationship between peer victimization and depressive symptoms. The reciprocal nature of this inconsistent association is in line with previous research (Hodges and Perry 1999; Vernberg 1990; McLaughlin et al. 2009).

The finding that peer victimization preceded increases in depressive symptoms is consistent with ego depletion models of stigma and social exclusion (Baumeister et al. 2005; Inzlicht et al. 2006). The effort to deal with peer victimization may deplete the resources necessary for self-regulation and reduce subsequent ability to effectively manage depressive symptoms, as suggested by McLaughlin et al. (2009). It should be noted that the reversed link from depressive symptoms to peer victimization was definitely less clear than the link from depressive symptoms to parental support. That depressive symptoms consistently preceded decreases in parental support, while less clear evidence was found for peer victimization, may be because peer victimization engages an overall evaluation of the person with depressive symptoms as a social stimulus, rather than a specific judgment of the person with depressive symptoms as an interaction partner (as applied to parental support). Previous findings have supported the idea that depressive symptoms do impact peer social supportive relations (e.g., Stice et al. 2004). Our findings may thus support inherently transactional interpersonal theories of depression (e.g., Coyne 1976; Coyne et al. 1991), in which mutual influence outcomes involve close interpersonal relationships.

In contrast to our hypothesis, victimization was not associated with expressive suppression over time. This suggests that victimized youth may not use suppression as a tool to prevent additional victimization. The fact that lower parental support was associated with more expressive suppression over time might provide support for the close interpersonal functions of suppression; that is, people may use suppression as a way of trying to manage relationship difficulties. Future studies may include different support providers to see whether lower support precedes the use of increased suppression across different support providers. Suppression requires cognitive control resources (Richards and Gross 2000), so it is possible that continuous suppression in broad victimization contexts requires too much self-regulation, but that youth are capable of short-term suppressing their display of emotion specifically in response to close persons from whom they experience less support. Future research should further examine the links between different types of interpersonal stressors and suppression over time.

### Limitations and Directions for Future Research

There are some limitations worth nothing. First, we did not measure peer support. As such, we cannot compare relationships with parents versus peers. Parental support was conceptualized as the adolescents’ perceptions about their parents in particular, while peer victimization corresponded to their perceptions about victimization by peers in general (who may or may not be friends or important people in the lives of these adolescents). Future research should include both close relationships with parents and peers. Second, we relied on a relatively healthy sample of adolescents who attended a high level of education and who were mostly from the same age range and same cultural background. In addition, attrition analyses showed that adolescents with somewhat lower levels of depressive symptoms and with some specific sociodemographic characteristic (e.g., intact families or younger adolescents) were overrepresented in the longitudinal sample. Consequently, findings from the present study may not generalize to less healthy populations or other populations that differ with regard to race, ethnicity, education, age, family structure, or depressive symptoms. Future research should further examine the link between depressive symptoms and suppression, as well as potential mediating variables, in less healthy populations, as well as healthy populations with different demographic characteristics (i.e., children or adults). Third, measurement waves were lagged by 1 year. Although this study was the first to offer insight into relationship variables mediating the relationship between depressive symptoms and expressive suppression, a longitudinal study including more measurements or a study using experience sampling would be particularly interesting to track and understand the potential mediating mechanisms. Finally, shared-method variance may have increased correlations among variables. Studies using methods other than self-reports (e.g., observational assessments or semi-structured interview to assess depression) may alleviate potential concerns regarding shared method variance and may provide more insight in underlying mechanisms.

### Conclusions

In sum, regarding longitudinal linkages, the present study further supports our initial work (Larsen et al. in press) suggestive of a unidirectional relationship from depressive symptoms to increased use of expressive suppression. Although negative reciprocal associations between depressive symptoms and parental support were generally supported, less evident and consistent reciprocal relationships were found between depressive symptoms and peer victimization. Some evidence was found for the negative prospective relationship from parental support to expressive suppression, while no significant prospective relationship was found between peer victimization and suppression. Related to the most central question of this investigation, parental support acted as a mediator in the prospective relationship from depressive symptoms to increased use of expressive suppression among adolescent girls. There was no evidence for a similar mediating role of peer victimization, or for other mediating models, because other longitudinal linkages necessary for mediation (i.e., longitudinal linkages between the dependent variable and the mediator, and between the mediator and the dependent variable) were not supported. Our findings suggest that parental support, but not peer victimization, is a mechanism explaining why girls who experience depressive symptoms report increased use of expressive suppression over time. This may suggest that increased use of suppression among girls with depressive symptoms may be a response to problems within close relationships. Theory-based interventions might target the interpersonal functions of suppression. However, before developing such interventions, future research should further test interpersonal mechanisms for explaining why girls with depressive symptoms who experience decreases in parental support report increased use of suppression and examine whether the mediating findings of parental support generalize across different support providers (i.e., peers, grandparents, siblings).
